# Oxidative stress and adipokine levels were significantly correlated in diabetic patients with hyperglycemic crises

**DOI:** 10.1186/s13098-019-0410-5

**Published:** 2019-02-06

**Authors:** Juan Li, Xingping Shen

**Affiliations:** 1 Department of Emergency, Zhongshan Hospital Xiamen University, Xiamen, 361004 Fujian China; 2 Department of Endocrinology, Zhongshan Hospital Xiamen University, Xiamen, 361004 Fujian China

**Keywords:** Hyperglycemic crises, Oxidative stress, Adipokines

## Abstract

**Background:**

To investigate the relationship between blood adipokine level and oxidative stress in diabetic patients with hyperglycemic crises before and after treatment.

**Methods:**

We measured superoxide dismutase (SOD) activity, malondialdehyde (MDA) content, total antioxidant capacity (TAC), and levels of 8-iso-prostaglandin F2α (8-iso-PGF2α), adiponectin, leptin, and resistin in 63 diabetic patients with hyperglycemic crises.

**Results:**

Prior to treatment, patients with hyperglycemic crises had significantly lower serum SOD activity, TAC, and adiponectin and leptin levels, and higher serum levels of MDA, 8-iso-PGF2α, and resistin compared with the healthy control individuals (all at P < 0.05). After treatment, SOD, TAC, adiponectin, and leptin levels increased significantly, while MDA, 8-iso-PGF2α, and resistin levels decreased significantly (all at P < 0.05) in the patients.

**Conclusions:**

Diabetic patients with hyperglycemic crises have increased oxidative stress, which is associated with serum adipokine abnormalities; improved oxidative stress after treatment suggests that oxidative stress may serve as target and/or indicator for the treatment of hyperglycemic crises.

## Introduction

Hyperglycemic crisis is a common acute complication of diabetes in patients with hyperglycemia [1, 2]. There are three types of hyperglycemic crisis: (a) diabetic ketoacidosis (DKA); (b) hyperosmolar hyperglycemic state (HHS) ([a] and [b] are two extremes of the same clinical syndrome); and (c) mixed syndrome (both DKA and HHS as a mixed state of acidosis and hyperosmolality). Basic and clinical studies have shown that hyperglycemic crises aggravate inflammation and increase the risks of myocardial infarction, heart failure, and cardiogenic shock. Acute hyperglycemia is therefore considered a risk factor for myocardial infarction in patients with type 2 diabetes [1, 2]. Hyperglycemia, an inducer of oxidative stress [3], causes the mitochondrial production of reactive oxygen species (ROS), leading to intracellular oxidative stress and insulin resistance [4]. In addition, hyperglycemia-induced ROS recruit inflammatory cells and stimulate the production of pro-inflammatory molecules such as cytokines, growth factors, and transcription factors [5]. Type 2 diabetic patients with hyperglycemic crises have significant oxidative stress, which exacerbates pathological damage to a variety of different organs [3, 6, 7]. Although the exact mechanism is yet to be elucidated, it is clear that oxidative stress plays an important role in the development of diabetic vascular disease.

Adipokines are a group of adipose tissue-derived bioactive molecules that are important in regulating diverse processes such as appetite, metabolism, fat distribution, insulin activity, and inflammation [8]. Adiponectin, leptin, and resistin are known adipokines. Adiponectin has anti-diabetic properties because it increases insulin sensitivity by reducing inflammation and oxidative stress [9, 10]. The insulin-sensitizing properties of adiponectin are important in maintaining the homeostasis of glucose and lipid metabolism [9]. Low adiponectin levels are closely associated with type 2 diabetes, insulin resistance, obesity, and cardiovascular risk [11]. Leptin is an adipose tissue-specific adipokine, and it acts as a sensing factor to regulate appetite, glucose homeostasis, energy metabolism, and immune cell functions [10, 12]. Leptin is a pro-inflammatory adipokine because it stimulates the activation of monocytes/macrophages and production of pro-inflammatory cytokines [12]. Leptin also increases ROS production and oxidative stress, which might explain the recently reported increases in cardiovascular disease among diabetic patients [12]. Elevated leptin levels are therefore considered a risk factor for cardiovascular disease. Resistin is an important mediator of cardiovascular and metabolic pathogenesis. High resistin levels are associated with inflammation [13], lead to vascular cell dysfunction, and induce ROS synthesis in various cells [14]. Indeed, a previous study found that poor clinical outcomes were associated with diabetic patients that had significantly elevated serum resistin levels [15].

Type 2 diabetic patients with hyperglycemic crises have often been diabetic for many years, and tend to have adhered poorly to recommended insulin therapies [16, 17]. Clinical manifestations in such patients typically include relatively short periods of abnormally elevated blood glucose levels. Preliminary studies have shown that oxidative stress has an increased effect on patients when blood glucose is drastically elevated. However, the relationship between the changes in adipokine levels and oxidative stress remains unclear. Thus, in this study we determined the relationship between in vivo oxidative stress markers and adipokine levels before and after insulin treatment in type 2 diabetic patients with hyperglycemic crises who self-discontinued insulin therapy due to various reasons.

## Subjects and methods

### Research subjects

Our study included 63 diabetic ketoacidosis (DKA) and non-ketotic hyperglycemia (NKH) patients (34 males and 29 females; mean age 50.68 ± 8.60 years; with a < 5-year course of type-2 diabetes and 3–14 day self-discontinuation of insulin analogue regimens). These patients were admitted to either the Endocrinology or the Emergency department of Zhongshan Hospital Xiamen University (Xiamen, Fujian Province, China) between June 2014 and December 2017. Criteria used to diagnose DKA were blood glucose > 13.9 mmol/L, blood pH < 7.3, blood HCO_3_ < 18 mmol/L, serum anion gap > 10 mmol/L, and ketonuria; criteria used to diagnose NKH were blood glucose > 22.4 mmol/L, blood pH > 7.3, blood HCO_3_ > 18 mmol/L, and absence of ketonuria [3, 18]. Patients with hyperglycemic crises had no signs of infection or of other disorders known to induce DKA and NKH. Patients with gastrointestinal hemorrhage, fever, endocrine disease, myocardial infarction, heart failure, heart disease, chronic obstructive pulmonary disease (COPD), renal insufficiency, pregnancy, or a smoking habit were excluded from our study. The therapeutic remission criteria were blood glucose < 13.9 mmol/L, blood pH > 7.3, blood HCO_3_ > 18 mmol/L, a normal anion gap, and a restoration of normal mental state. The participants in the matching control group were 30 healthy individuals who were recruited during their visit to the Physical Examination Center of the Zhongshan Hospital Xiamen University. These control subjects were free of heart, brain, kidney, endocrine, and metabolic diseases. The major characteristics (gender, age, body weight, and BMI) in the control group were closely matched to those in the case groups (Table 1). In the study, 5 mL fasting venous blood was collected from each participant in the morning. Clinical information and laboratory test results for all participants were recorded. Our research protocol was approved by the Ethics Committee of Zhongshan Hospital Xiamen University. All research subjects signed an informed written consent before participating in our study.

**Table 1:** General characteristics of study participants (x ± s)

	Control	DKA		NKH	
		Before treatment	After treatment	Before treatment	After treatment
No. cases (male/female)	30 (14/16)	32 (18/14)		31 (16/15)	
Age (years)	49.85 ± 8.72	51.45 ± 11.96		50.63 ± 10.06	
BMI (kg/m^2^)	25.87 ± 5.91	24.19 ± 7.83		24.99 ± 9.07	
WHR	0.87 ± 0.09	0.86 ± 0.04		0.88 ± 0.10	
Body temperature (°C)	36.20 ± 3.33	37.03 ± 6.89	36.95 ± 7.75	37.10 ± 7.12	36.84 ± 6.83
Systolic pressure (mmHg)	128 ± 18	126 ± 20	130 ± 18	126 ± 22	128 ± 16
Diastolic pressure (mmHg)	78 ± 9	76 ± 8	78 ± 12	76 ± 10	77 ± 11
White blood cell count (× 10^6^)	7.82 ± 1.16	16.94 ± 6.57^a^	8.65 ± 4.28^b^	14.93 ± 7.22^a^	8.45 ± 6.58^b^

DKA: diabetic ketoacidosis group; NKH: non-ketotic hyperglycemia group; control group: healthy individuals

^a^P < 0.05, compared with controls

^b^P < 0.05, compared with the same group before treatment

### Methods

#### Treatment plan

Patients were administered with insulin (0.1 U/kg/h) via a micropump as previously described [6]. The amount of insulin given was adjusted accordingly based on changes in blood glucose. Patients were rehydrated and given other symptomatic treatments as necessary. All DKA and NKH patients received identical gastrointestinal and/or parenteral nutritional support. The therapeutic remission criteria were blood glucose < 13.9 mmol/L, blood pH > 7.3, blood HCO_3_ > 18 mmol/L, a normal anion gap, and a restoration of normal mental state.

#### Blood sample collection and outcome measurement

Arterial and venous blood samples were collected from each participant at the time of hospital admission and 72 h after disease remission. Blood glucose was estimated with the glucose oxidase method; HbA1c was detected with high performance liquid chromatography; blood leukocytes were counted using a 5-part differential hematology analyzer (Beckman Coulter, Inc. Brea, CA, USA); Creatinine (Cr) was measured with the alkaline picrate method; blood urea nitrogen was measured with the enzyme coupling ratio method; and electrolytes were measured using enzymatic analysis. Blood gas values were determined using a blood gas analyzer (AVL COMPACT3; Roche Diagnostics GmBH, Mannheim, Germany). GADab was detected with an enzyme-linked immunosorbent assay (ELISA) kit (Biomerica Inc., Irvine, CA, USA). C-peptides were detected with an electrochemiluminescence immunoassay. 8-iso-PGF_2α_ was quantified with an ELISA kit (Nanjing Jiancheng Bioengineering Institute, Nanjing, Jiangsu Province, China). SOD activity, MDA content, and TAC were measured with respective analytical kits (Nanjing Jiancheng Bioengineering Institute). Adiponectin, leptin, and resistin assays were performed with ELISA kits (Boster Biological Technology Co., Ltd., Wuhan, Hebei Province, China). In addition, all participants were given routine examinations, including urinalysis, X-rays, and electrocardiograms.

#### Measurement of body mass index (BMI) and waist circumference/hip ratio (WHR)

Once the conditions of the DKA and NKH patients stabilized, their heights (in cm, to 0.1 cm) and weights (wearing underwear only; in kg, to 0.1 kg) were measured. We also measured waist circumference (WC) at the midpoint between the lower rib and upper border of the iliac crest, and hip circumference at the level of the greater trochanter of the femur (both in cm, to 0.1 cm). The following equations were used to calculate BMI and WHR: BMI = bodyweight (kg)/height (m)^2^ and WHR = waist circumference/hip circumference.

#### Statistical analysis

SPSS 15 (SPSS Inc., Chicago, IL, USA) was used for statistical analyses. All data were presented as means ± standard deviations (*x *± *s*). We used paired Student’s *t* test to compare measurements before and after treatment. Between-group comparison was conducted using the non-paired Student’s *t* test and correlation analysis was conducted using the Pearson’s correlation test. Significance was set at P < 0.05.

## Results

### General clinical data

Patients with hyperglycemic crises (both DKA and NKH) had significantly higher blood leukocyte counts, blood glucose levels, and HbA1c levels than did the healthy controls (all at P < 0.05). After treatment, the blood leukocyte counts in the diabetic patients were significantly lower than before treatment (P < 0.05), and were not significantly different from the counts in the control group. In the treated DKA patients, blood glucose levels were significantly lower, anion gaps were significantly smaller, and blood pH and HCO_3_^−^ were significantly higher, as compared to levels before treatment (all at P < 0.05). Blood glucose levels in the NKH patients were also significantly lower after treatment (P < 0.05). In both the DKA and NKH patients, C-peptide levels were significantly lower than controls, irrespective of treatment (P < 0.05, Tables 1 and 2).

**Table 2:** Biochemical features of diabetic patients with hyperglycemic crises and the healthy controls (x ± s)

	Control	DKA		NKH	
		Before treatment	After treatment	Before treatment	After treatment
Blood glucose (mmol/L)	5.18 ± 2.76	23.09 ± 9.43^a^	7.26 ± 4.60^b^	25.86 ± 11.66^a^	7.95 ± 4.38^b^
HbA1c (%)	5.60 ± 1.28	13.87 ± 9.51^a^		14.78 ± 6.28^a^	
APH	7.39 ± 2.11	7.19 ± 4.78^a^	7.37 ± 3.15^b^	7.38 ± 3.01	7.39 ± 4.64
HCO_3_^−^(mmol/L)	24.12 ± 2.20	12.35 ± 1.98^a^	23.85 ± 2.67^b^	23.43 ± 2.62	23.55 ± 3.25
Ketones in urine	−	+	−	−	−
Effective osmolality (mmol/L)	284.20 ± 14.71	280.43 ± 16.04	282.76 ± 14.39	290.62 ± 18.71	291.53 ± 20.35
Anion gaps (mmol/L)	12.11 ± 3.10	29.15 ± 5.88^a^	13.56 ± 4.04^b^	12.87 ± 4.80	11.38 ± 5.18
C-peptide (nmol/L)	0.76 ± 0.07	0.34 ± 0.05^a^	0.35 ± 0.09^a^	0.41 ± 0.09^a^	0.43 ± 0.09^a^

DKA: diabetic ketoacidosis group; NKH: non-ketotic hyperglycemia group; control group: healthy individuals

^a^P < 0.05, compared with controls

^b^P < 0.05, compared with the same group before treatment

### Oxidative stress and adipokine levels in patients with hyperglycemic crises before and after treatment

Before treatment, diabetic patients with hyperglycemic crises had significantly lower levels of SOD activity, TAC, adiponectin, and leptin, and significantly higher levels of MDA, 8-iso-PGF_2α_, and resistin as compared to the control group (all at P < 0.05). After treatment, levels of SOD activity, TAC, adiponectin, and leptin increased significantly in the diabetic patients, while levels of MDA, 8-iso-PGF_2α_, and resistin decreased significantly (all at P < 0.05). However, after treatment, levels of SOD activity, TAC, and adiponectin in the patients with hyperglycemic crises remained significantly lower), while levels of MDA, 8-iso-PGF_2α_, and resistin remained significantly higher than the control group (all at P < 0.05; Table 3).

**Table 3:** Oxidative stress and adipokines in diabetic patients with hyperglycemic crises before and after treatment and the healthy controls (x ± s)

	Control	DKA		NKH	
		Before treatment	After treatment	Before treatment	After treatment
SOD (kU/L)	89.56 ± 14.16	48.76 ± 18.33^a^	69.52 ± 16.31^ab^	53.52 ± 14.70^a^	70.35 ± 13.80^ab^
MDA (µmol/L)	5.23 ± 2.57	11.37 ± 3.66^a^	7.64 ± 2.90^ab^	8.57 ± 3.01^a^	6.07 ± 2.67^ab^
TAC (kU/L)	29.51 ± 11.49	20.39 ± 10.61^a^	24.27 ± 10.05^ab^	20.95 ± 12.41^a^	23.02 ± 11.23^ab^
8-Iso-PGF2α (µg/L)	6.70 ± 2.33	18.42 ± 5.78^a^	15.14 ± 6.21^ab^	20.90 ± 4.70^a^	13.44 ± 6.71^ab^
Adiponectin (µg/L)	9.89 ± 1.63	5.77 ± 1.55^a^	7.12 ± 1.63^ab^	5.23 ± 1.11^a^	8.06 ± 1.56^ab^
Leptin (µg/L)	8.91 ± 1.73	6.07 ± 2.45^a^	8.45 ± 2.13^b^	5.91 ± 1.07^a^	8.78 ± 2.65
Restistin (µg/L)	3.21 ± 1.07	8.35 ± 1.83^a^	6.24 ± 1.50^ab^	9.23 ± 2.06^a^	7.14 ± 1.99^ab^

DKA: diabetic ketoacidosis group; NKH: non-ketotic hyperglycemia group; control group: healthy individuals

^a^P < 0.05, compared with controls

^b^P < 0.05, compared with the same group before treatment

### Correlation analyses

In the pre-treatment diabetic patients with hyperglycemic crises, adiponectin and 8-iso-PGF_2α_ were negatively correlated (r = − 0.37, P < 0.05), as were leptin and MDA (r = − 0.34, P < 0.05). In the DKA patients after treatment, adiponectin was negatively correlated with MDA (r = − 0.31, P < 0.05) and 8-iso-PGF_2α_ (r = − 0.29, P < 0.05). In the NKH patients after treatment, MDA and adiponectin were also negatively correlated (r = − 0.43, P < 0.05). In all patients after treatment, 8-iso-PGF_2α_ was negatively correlated with leptin (r = − 0.37, P < 0.05) and with MDA (r = − 0.35, P < 0.05). In all patients, resistin and MDA were positively correlated both before (r = 0.30, P < 0.05) and after (r = 0.38, P < 0.05) treatment (Table 4).

**Table 4:** Simple correlation between oxidative stress and adipokine in diabetic patients with hyperglycemic crises

	Hyperglycemic crises (before treatment)				Hyperglycemic crises (after treatment)				DKA (after treatment)				NKH (after treatment)			
	8-Iso-PGF2α		MDA		8-Iso-PGF2α		MDA		8-Iso-PGF2α		MDA		8-iso-PGF2α		MDA	
	r	P	r	P	r	P	r	P	r	P	r	P	r	P	r	P
Adiponectin	− 0.370	0.0412	− 0.213	0.0766	− 0.286	0.0637	− 0.315	0.0853	− 0.293	0.0262	− 0.310	0.0433	− 0.181	0.1030	− 0.432	0.0280
Leptin	− 0.283	0.0674	− 0.341	0.0376	− 0.373	0.0273	− 0.353	0.0393	− 0.126	0.2183	− 0.210	0.1821	− 0.196	0.3185	− 0.266	0.1060
Restistin	0.107	0.2110	0.302	0.0283	0.285	0.0973	0.383	0.0075	0.112	0.1083	0.276	0.095	0.197	0.0894	0.202	0.0961

## Discussion

Dysregulated redox balance in local or systemic antioxidant defense system is involved in the pathogenesis of diabetes and its complications. Diabetes is often accompanied by an increased oxidative stress due to the excessive functional decline of the antioxidant defense system and the consequently increased production of free radicals. Thus, identification of sensitive biomarkers for the redox status will be very helpful for the assessment of diabetic complications and treatment effect. However, because a wide variety of interactive antioxidants may be produced, accurate detection of any single antioxidant is difficult. 8-iso-PGF_2α_, a non-enzymatic product of arachidonic acid metabolism, is considered the most reliable marker for lipid peroxidation and its concentration in blood are often used as an accurate index of human oxidative stress. Here, we used this metric along with multiple other measurements to produce a more comprehensive and potentially more accurate assessment of oxidative stress.

MDA is a toxic lipid peroxidation metabolite that has been considered a marker for the cellular damages caused by oxygen free radicals [19], while SOD is a key antioxidant enzyme in the body that plays an important role in reducing the damage caused by reactive oxygen metabolites. TAC represents the total peroxide damage caused by naturally occurring low-molecular weight enzymatic free radical scavengers, and reflects the effect of this damage on the enzymatic and non-enzymatic antioxidant balance in the body [20]. Increased generation of ROS in tissues and body fluids has been shown to reduce TAC [21].

Here, we identified significant differences in 8-iso-PGF_2α_ level, SOD activity, MDA content, and TAC in diabetic patients with hyperglycemic crises as compared to the healthy controls, consistent with previous studies [3, 6]. Hyperglycemia increases superoxide anion production in the neutrophils and monocytes, increasing the expression of the p47^phox^ subunit of the NADPH oxidase complex, which may in turn increase the conversion of molecular oxygen to superoxidated anions [22]. Reduced TAC in patients with hyperglycemic crises was associated with insufficient TAC tissue reserves, and a decreased ability to regenerate TAC. When HbA1c levels are high, blood glucose fluctuations can cause oxidative stress [23]. In the diabetic patients with hyperglycemic crises in our study, HbA1c levels were > 13%. Acute hyperglycemia and blood glucose fluctuations reduce the expression of genes that alleviate the toxic effects of free radicals, thereby increasing oxidative stress [7, 23, 24].

Insulin reduces oxidation and acute inflammation comprehensively and effectively [25, 26]. Here, insulin therapy and rehydration relieved hyperglycemia in patients with hyperglycemic crises. In these patients, SOD activity, MDA content, TAC, and 8-iso-PGF_2α_ levels were significantly improved after treatment because insulin significantly reduces the expression of the p47^phox^ subunit of the NADPH oxidase complex, directly inhibiting NADPH oxidase [25, 27]. Insulin reduces the expression of gp91phox, a critical component of NADPH oxidase, leading to decreased production of ROS [25, 27].

Although indices of oxidative stress in the diabetic patients improved after hyperglycemic crisis treatment, these indices were still significantly different from those of the control group. This is probably because hyperglycemic crises have a relatively short remission time, but these patients had both relatively long courses of diabetes and acute blood glucose fluctuations during treatment [7]. As blood glucose fluctuations are independent prognostic factors of various critical illnesses, our results suggested that the minimization of blood glucose fluctuations in diabetic patients with hyperglycemic crises during recovery would reduce oxidative stress. Indeed, oxidative stress during hyperglycemic crises in diabetics might be best understood as a series of pathological events caused by the hyperglycemia-induced imbalance between pro-oxidant and antioxidant mechanisms.

Decreased serum adiponectin is an independent indicator of type 2 diabetes progression risk [28]. Here, adiponectin levels in patients with hyperglycemic crises before treatment were significantly lower than those in the control group, which is associated with the long-term unsatisfactory control of their diabetes. Adiponectin levels increased in patients with hyperglycemic crises following the treatment but were still lower than those in the control group, indicating that reduced blood glucose levels were associated with increased serum adiponectin. After treatment, increased serum adiponectin reduced blood sugar by inhibiting hepatic gluconeogenesis and hepatic glucose production rate [29, 30]. Adiponectin levels in patients with hyperglycemic crises both pre- and post-treatment were negatively correlated with oxidative stress. This might be because oxidative stress decreases adiponectin production by inhibiting peroxisome proliferator-activated receptor gamma (PPARγ) mRNA expression, decreasing nuclear PPARγ content, increasing NADPH oxidase activity, and reducing antioxidant enzyme activity [31]. With the relief of the hyperglycemic crisis, the blood glucose of the patient declines, and adiponectin can inhibit oxidative stress through the cAMP/PKA (protein kinase)-dependent pathway [32].

Consistent with a previous study [33], leptin levels in the pre-treatment diabetic patients with hyperglycemic crises were significantly lower than those in the control group; leptin levels increased significantly after hyperglycemia treatment. During hyperglycemic crises, high levels of counter-regulatory hormones (glucagon, cortisol, growth hormones, and catecholamines) are present. Cortisol strongly stimulates leptin production as shown in both the in vitro and in vivo studies [34], but this effect is negated by metabolic acidosis, dehydration, caloric depletion, fasting, and hunger in patients with hyperglycemic crises. Leptin secretion depends on glucose utilization in adipocytes, but the efficiency of glucose use is significantly attenuated in adipocytes during hyperglycemic crises. This decline in leptin levels leads to the elevation of corticotropin releasing factor/adrenocorticotropic hormone (CRF/ACTH), corticosterone, gluconeogenesis, and ketogenesis. These effects aggravate DKA metabolic abnormalities [35]. Leptin is an effective ventilation stimulant that acts on central respiratory control nuclei [12], and high leptin levels are associated with an increase in minute ventilation [36]. Therefore, it is possible that the commonly observed shortness of breath in diabetic patients with hyperglycemic crises is related to high leptin levels.

Insulin plays an important role in the regulation of leptin gene expression and protein synthesis. Insulin is clearly depleted during hyperglycemic crises. In diabetic patients with hyperglycemic crises, insulin therapy relieves the hyperglycemic crises and significantly increases serum leptin, suggesting that insulin may stimulate increased leptin gene transcription, gene expression, and synthesis leading to the increased presence of blood leptin levels [37]. Insulin may also elevate blood leptin levels through increasing secretion of glucocorticoids, which stimulate leptin secretion and also enhance glucose utilization in adipocytes [33]. As leptin levels increase, hepatic gluconeogenesis and ketogenesis decrease with the hypothalamic–pituitary–adrenal axis mediated systemic lipolysis, reversing the hyperglycemic crisis [35, 38]. Here, elevated leptin levels in patients with hyperglycemic crises before and after treatment were associated with oxidative stress. It is probable that leptin has an important role in the induction and regulation of the redox system, as hyperleptinemia during hyperglycemic crises induces oxidative stress in various organs and tissues [12], and the production of ROS by phagocytic and non-phagocytic cells is induced by leptin through the activation of NADPH oxidase [39]. High resistin levels are a risk factor for cardiovascular disease and all-cause mortality in patients with type 2 diabetes [40]. Here, the pre-treatment resistin levels of diabetic patients with hyperglycemic crises were significantly higher than those of the control group. After treatment, resistin levels decreased significantly in the diabetic patients, but remained significantly higher than those of the controls. In addition, resistin levels pre- and post-treatments were both positively correlated with MDA. Hyperglycemic crises cause substantial inflammation [6], leading to the production of excess resistin by activated neutrophils [41]. Resistin induces ROS synthesis through protein kinase C epsilon (PKCε)-mediated NOX activation, further increasing oxidative stress [15]. Here, high resistin levels, inflammation state, and the aggravation of oxidative stress were observed in diabetic patients with hyperglycemic crises before insulin before treatment.

Diabetic patients with hyperglycemic crises, who often have comorbidities such as serious cardiovascular diseases and other high-risk factors, are highly susceptible to various additional cardiovascular and cerebrovascular complications. Our results suggest that the timely application of intensive insulin therapy to diabetic patients during hyperglycemic crises may be helpful in steadily reducing blood sugar, lowering oxidative stress, correcting dysregulation of adipokine production, and ultimately reducing the risk of cardiovascular and cerebrovascular complications. However, a limitation of the current study is the relatively small sample size. Thus, future studies with larger sample sizes are needed to validate our results.

## Abbreviations

SOD: superoxide dismutase
MDA: malondialdehyde
TAC: total antioxidant capacity
8-iso-PGF2α: 8-iso-prostaglandin F2α
ROS: reactive oxygen species
DKA: diabetic ketoacidosis
NKH: non-ketotic hyperglycemia
PPARγ: peroxisome proliferator-activated receptor gamma
CRF/ACTH: corticotropin releasing factor/adrenocorticotropic hormone
